# Supplementary material for: HPF1 Regulates Pol β Efficiency in Nucleosomes via the Modulation of Total Poly(ADP-Ribose) Synthesis
Source: Int J Mol Sci. 2025 Feb 20;26(5):1794. doi: 10.3390/ijms26051794 (PMC11898694; doi:10.3390/ijms26051794)
Supplement: Supplementary file 1 [file ijms-26-01794-s001.zip › Supplementary.pdf]

Supplementary Materials

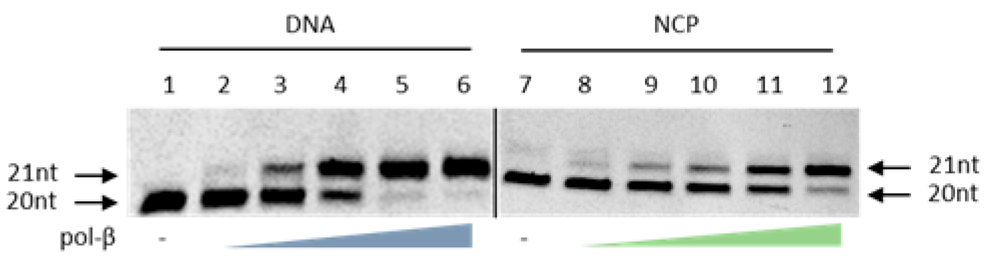

Figure S1. The activity of the DNA polymerase β in single-nucleotide gap-filling reactions. Fluorescent electropherogram after PAGE of probes under denaturing conditions. Reactions were performed in the presence of 100 μM dTTP. Lanes 1 – 6 – naked DNA; lanes 7 – 12 – NCP. Probes 2 – 6 contained pol β in the concentrations of 0.03 nM, 0.1 nM, 0.3 nM, 1.0 nM, 3.0 nM; probes 8 – 12 contained pol β in the concentrations of 0.01 μM, 0.03 μM, 0.06 μM, 0.1 μM, 0.3 μM. Lane 1 and 7 did not contain DNA pol β.

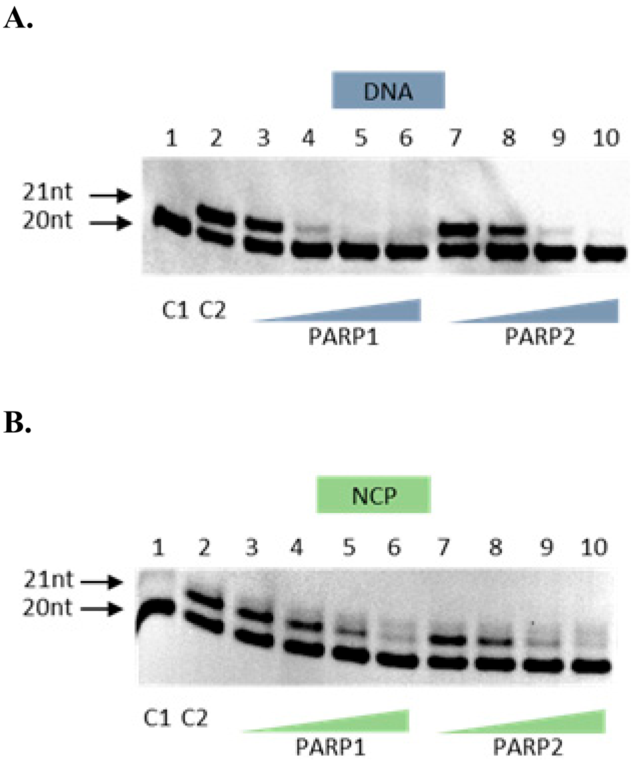

Figure S2. The PARPs influence pol β activity under single-nucleotide gap-filling reaction conditions. Fluorescent electropherogram after PAGE of probes under denaturing conditions. Panel A – the reactions using DNA. Reactions performed in the presence of 0.3 nM pol β. Panel B – the reaction using NCP. Reaction performed in the presence of 60 nM pol β. The concentration of PARP1 or PARP2 varied in a diapason of 10 – 300 nM. C1 – control probe without pol β. C2 – control probe with pol β.

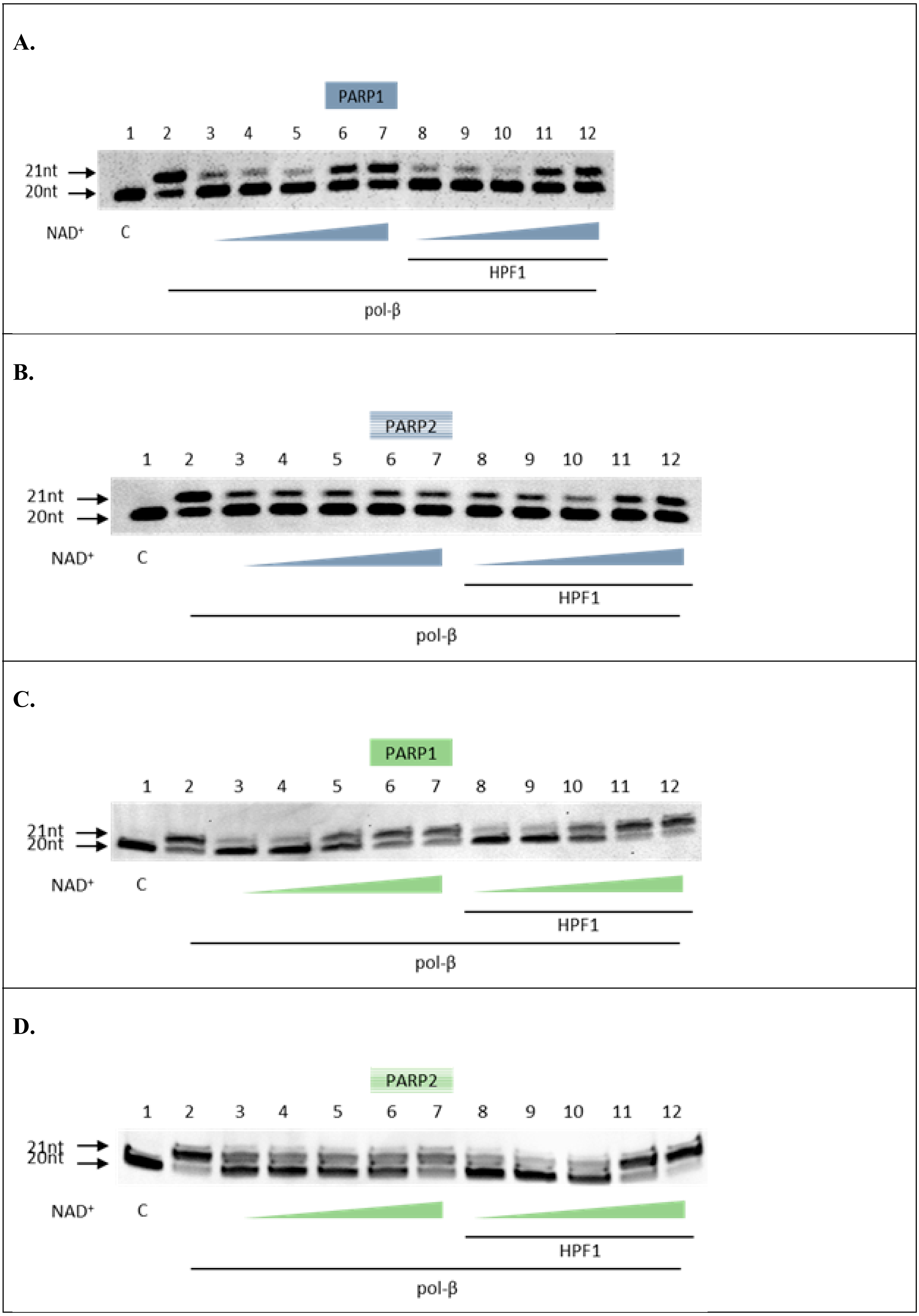
A.
PARP1
1 2 3 4 5 6 7 8 9 10 11 12
21nt
20nt
NAD+
C
HPF1
pol-β
B.
PARP2
1 2 3 4 5 6 7 8 9 10 11 12
21nt
20nt
NAD+
C
HPF1
pol-β
C.
PARP1
1 2 3 4 5 6 7 8 9 10 11 12
21nt
20nt
NAD+
C
HPF1
pol-β
D.
PARP2
1 2 3 4 5 6 7 8 9 10 11 12
21nt
20nt
NAD+
C
HPF1
pol-β

Figure S3. The efficiency of gap-filling DNA synthesis catalyzed by pol β using DNA or NCP depending on the PARylation and HPF1. Fluorescent electropherogram after PAGE of probes under denaturing conditions. Reactions were performed in the presence of 100 µM dTTP. Panels A and B – reactions using DNA, panels C and D - reactions using NCP. Reactions were performed in the presence of either PARP1 or PARP2 as it is mentioned in the figure. The concentrations of NAD^+ were titrated from 0,1 µM to 100 µM in lanes 3-7 and 8-12. The probes 8 – 12 were supplemented with HPF1. C – control probe without pol β. Lane 2 - dTMP incorporation by pol β alone.

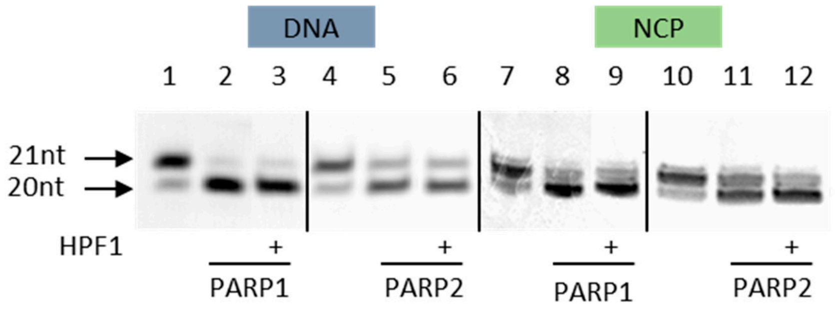

Figure S4. The influence of PARPs and HPF1 on the efficiency of gap-filling DNA synthesis catalyzed by pol β using DNA and NCP. Fluorescent electropherogram after PAGE of probes under denaturing conditions. Reactions were performed in the presence of 100 µM dTTP. Lanes 1, 4, 7, and 10 - the dTMP incorporation by pol β alone; lanes 2, 5, 8, and 11 - the dTMP incorporation by pol β and consequent PARP; lanes 3, 6, 9, and 12 - the dTMP incorporation by pol β and consequent PARP supplemented with HPF1.

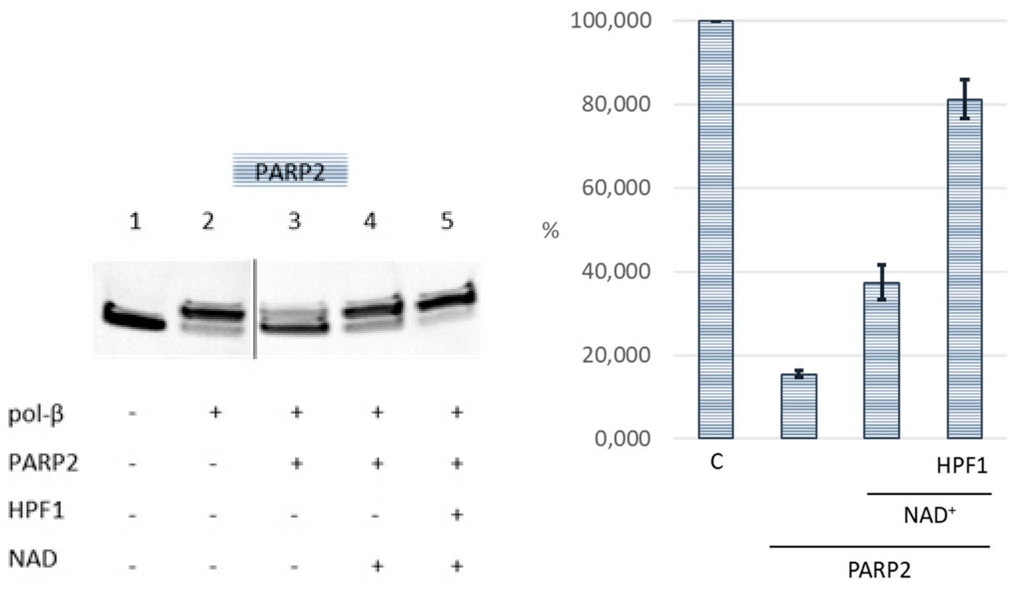

Figure S5. The influence of PARP2 and HPF1 on the efficiency of gap-filling DNA synthesis catalyzed by pol β using DNA. Left panel - fluorescent electropherogram after PAGE of probe under denaturing conditions. Reactions were performed in the presence of 100 µM dTTP. Right panel – quantitative representation of the dTMP incorporation efficiency.

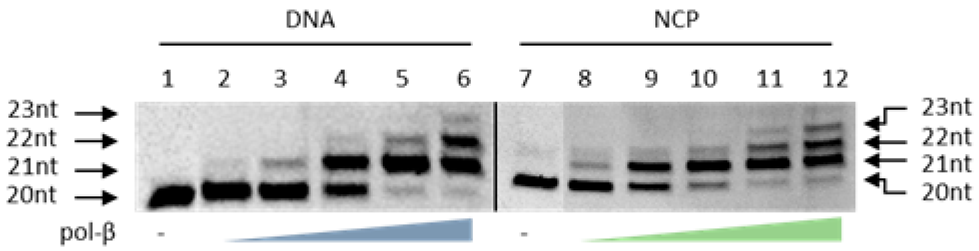

Figure S6. The activity of the DNA polymerase β in the DNA strand-displacement. Fluorescent electropherogram after PAGE of probes under denaturing conditions. Reaction performed in presence of four dNTP in total concentration of 400 µM. Lanes 1 – 6 – reactions using naked DNA; lanes 7 – 12 – reactions using NCP. Probes 2 – 6 contained pol β in the concentrations of 0.03 nM, 0.1 nM, 0.3 nM, 1.0 nM, 3.0 nM; probes 8 – 12 contained pol β in the concentrations of 0.03 µM, 0.1 µM, 0.3 µM, 1.0 µM, 3.0 µM. Probes 1 and 7 are controls and did not contain DNA pol β.

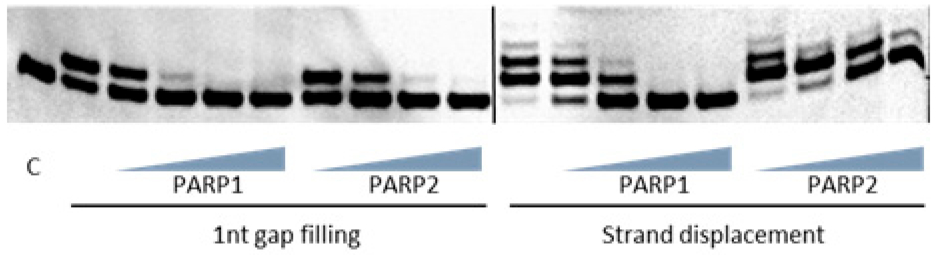

A. DNA

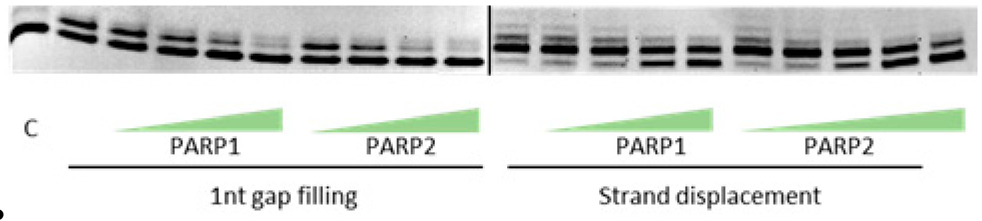

B. NCP

Figure S7. The PARPs influence pol β activity under strand-displacement reaction conditions. Fluorescent electropherogram after PAGE of probes under denaturing conditions. Panel A – the reactions using DNA were performed in the presence of 3.0 nM pol β. The concentrations of PARP1 or PARP2 were varied in a diapason of 10 – 300 nM. Panel B – the reactions using NCP were performed in the presence of 1 µM pol β. The concentrations of PARP1 were varied in a diapason of 30 nM – 1 µM, the concentrations of PARP2 were varied in a diapason of 10 nM – 1 µM. C1, C2 – probes without of pol β.

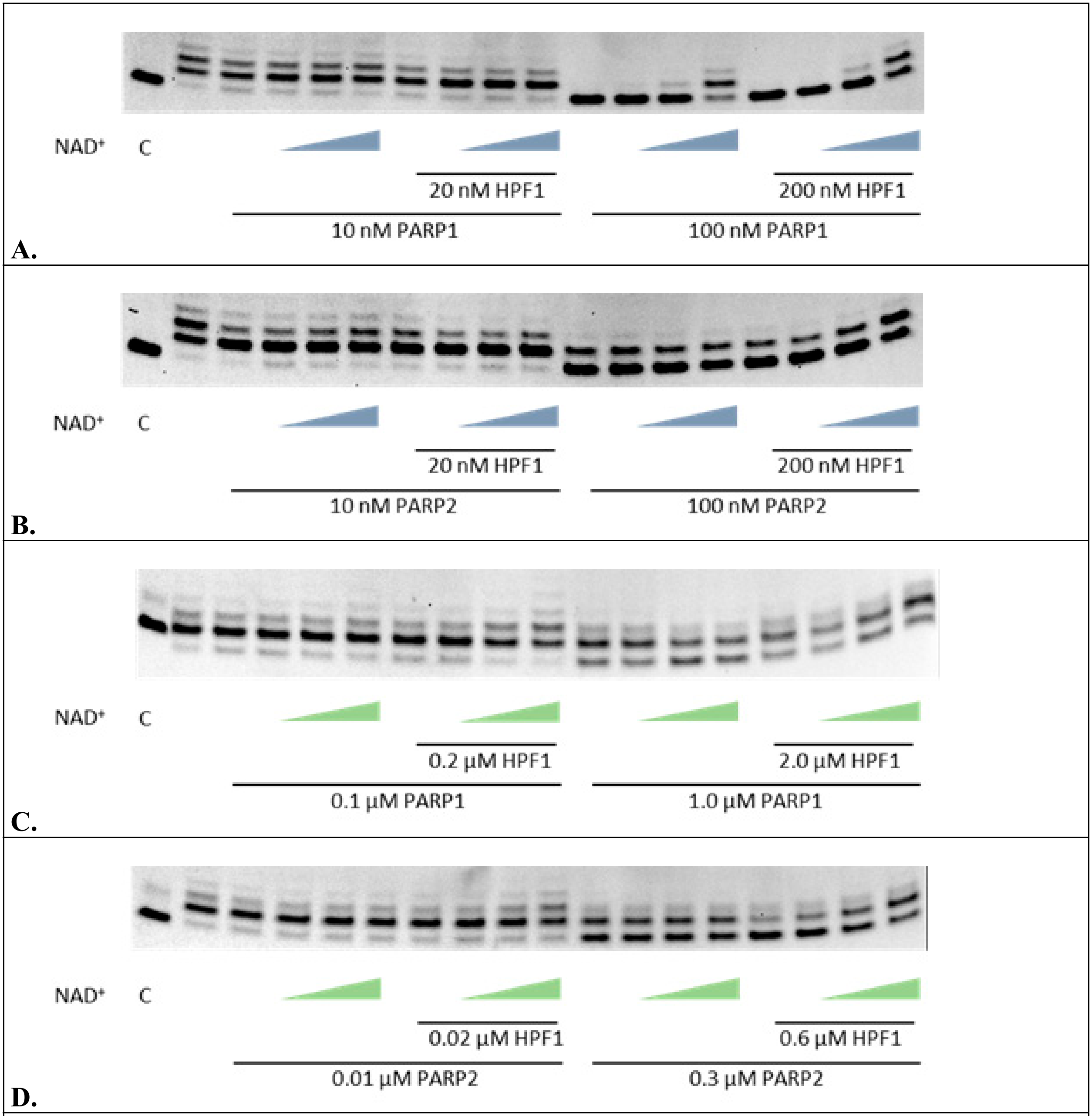

Figure S8. The efficiency of strand-displacement DNA synthesis catalyzed by pol β using DNA and NCP depending on the PARylation. Fluorescent electropherogram after PAGE of probes under denaturing conditions. Panels A and B – reactions using DNA, panels C and D - reactions using NCP. Reactions were performed in the presence of 400 μM dNTP and either PARP1 or PARP2 as it is mentioned in the figure. The concentrations of NAD^+ were titrated from 0,1 μM to 100 μM, lanes 3-7 and 8-12. The probes 8 – 12 were supplemented with HPF1.

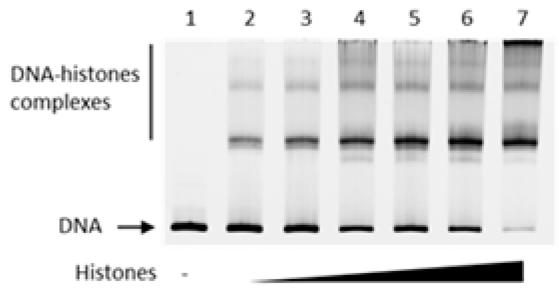

A.

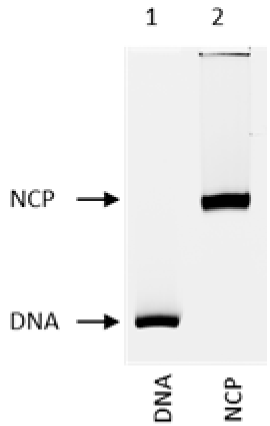

B.

Figure S9. The NCP reconstruction. Fluorescent electropherogram after probe separation under non-denaturing conditions. Panel A - preliminary NCP reconstruction. The molar ratio of core histone to DNA varied from 1:0.4 (lane 2) to 1:1.4 (lane 7). Lane 1 – free DNA. Panel B - preparative NCP reconstruction. Lane 1 – free DNA, lane 2 – reconstituted NCP.
